# Spurious electroencephalographic activity due to pulsation artifact in the depth of anesthesia monitor

**DOI:** 10.1186/s40981-021-00441-z

**Published:** 2021-04-17

**Authors:** Kotoe Kamata, Tarmo Lipping, Arvi Yli-Hankala, Ville Jäntti, Masanori Yamauchi

**Affiliations:** 1 Department of Anesthesiology and Perioperative Medicine, Tohoku University School of Medicine, 2-1 Seiryo-machi, Aoba-ku, Sendai-shi, Miyagi 980-8575 Japan; 2 Faculty of Information Technology and Communication, Tampere University, Pohjoisranta 11, 28100 Pori, Finland; 3 Department of Anesthesia, Tampere University Hospital, Elämänaukio 2, 33520 Tampere, Finland; 4 Faculty of Medicine and Health Technology, Tampere University, Kalevantie 4, 33100 Tampere, Finland; 5 Department of Clinical Neurophysiology, Seinäjoki Central Hospital, Hanneksenrinne 7, 60220 Seinäjoki, Finland

**Keywords:** Artifact, Depth of anesthesia, Electroencephalogram, Intraoperative, Monitoring, Pulse wave

## Abstract

**Background:**

The depth of anesthesia (DOA) is estimated based on the anesthesia-induced electroencephalogram (EEG) changes. However, the surgical environment, as well as the patient him/herself, generates electrical interferences that cause EEG waveform distortion.

**Case presentation:**

A 52-year-old patient required general anesthesia due to the right femur necrotizing fasciitis. He had no history of epilepsy or head injury. His cardiovascular status was stable without arrhythmia under propofol and remifentanil anesthesia. The DOA was evaluated with Root® with SedLine® Brain Function Monitoring (Masimo Inc, Irvine, CA). The EEG showed a rhythmic, heart rate time-locked pulsation artifact, which diminished after electrode repositioning. Offline analysis revealed that the pulse wave-like interference in EEG was observed at the heart rate frequency.

**Conclusions:**

We experienced an anesthesia case that involves a pulsation artifact generated by the superficial temporal artery contaminating the EEG signal. Numerous clinical conditions, including pulsation artifact, disturb anesthesia EEG.

## Background

Several kinds of monitors have been developed to measure the depth of anesthesia (DOA). These monitors calculate indices from a large electroencephalogram (EEG) database using proprietary software [1–3]. Previous reports show inaccurate EEG-derived indices caused by waveform distortion. High facial muscle activities and electrical noise are the leading causes of artifacts on anesthesia EEG [4]. Here we experienced an anesthesia case that involves a pulsation artifact generated by the superficial temporal artery (STA) contaminating EEG signal.

## Case presentation

The study was approved by the Ethics Committee of Tohoku University School of Medicine (No. 21334). The written informed consent was obtained from the patient for publication of this case report and any accompanying images. A 52-year-old male (180 cm, 84.2 kg) suffered from septic shock due to right femur necrotizing fasciitis. He had no history of epilepsy or head injury. Preoperative brain computed tomography and chest X-ray revealed no abnormalities. Electrocardiogram (ECG) showed a normal sinus rhythm. Five days after the initial operation of the right below-knee amputation, debridement was planned for local infection control. He was classified as American Society of Anesthesiologists (ASA) Physical Status Class 3E.

**Fig. 1:**
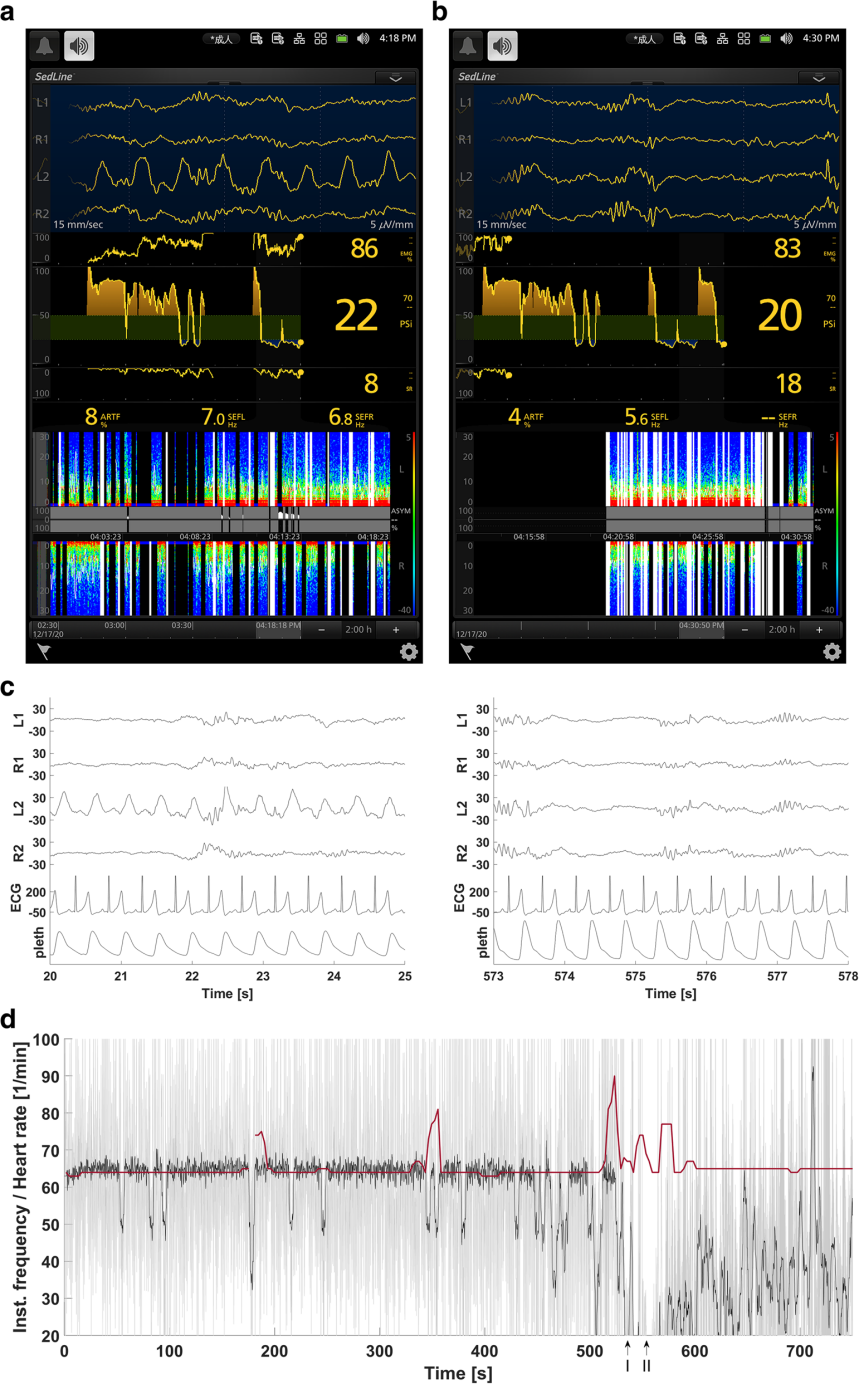
Normalization of electroencephalography after L2 electrode repositioning. **a** A snapshot of the Root® with SedLine® Brain Function Monitoring (Masimo Inc, Irvine, CA) is presented. A rhythmic, heart rate time-locked pulsation artifact is overlying L2. **b** Following L2 electrode repositioning, a pulsation artifact has completely disappeared. All four electroencephalogram (EEG) traces show a burst-suppression pattern. **c** Two 10-s samples (left and right) of four EEG channels (L1, R1, L2, and R2), electrocardiogram (ECG), and plethysmography (pleth) show how the waveform of L2 changes after electrode replacement. The synchronization between ECG/pleth versus EEG is not perfect due to separate standalone monitoring devices, but it can be seen that the pace of the low fluctuation in L2 EEG is similar to that of the heart rate and pleth. The units of EEG and ECG signals are microvolts. **d** Heart rate (red) and instantaneous frequency of the low-frequency component of L2. Both sample-by-sample estimate and smoothed version over a 4-s window are shown in black color. The time axis values correspond to those in **c** above. The Roman numerals I and II indicate the timepoints of making the start of the electrode replacement (I) and recovery of the EEG signal (II), respectively

Under intravenous sedation with dexmedetomidine (12 μg h^−1^) and fentanyl (100 μg h^−1^), the patient successfully adapted to a mechanical ventilator with endotracheal intubation. Besides standard ASA monitoring, invasive blood pressure was continuously recorded by an automated anesthesia recording system (PRM-7000; Nihon Kohden Co., Tokyo, Japan). The DOA was evaluated with Root® with SedLine® Brain Function Monitoring Version 1.8.1.4i (Masimo Inc, Irvine, CA), as a standalone monitor. Surgical anesthesia was induced with additional propofol (500 mg h^−1^), remifentanil (1 mg h^−1^), and rocuronium (50 mg). Continuous infusion of phenylephrine (1 mg h^−1^) controlled the intraoperative hemodynamics while maintaining his mean arterial pressure within 25% of the baseline value (i.e., hypotension-related EEG abnormalities were minimized). His heart rate was stable at around 64 bpm without arrhythmia. The EEG showed a rhythmic, heart rate time-locked pulsation artifact over one of four traces, L2, when there was a momentary pause in the electrocautery interferences (Fig. 1a). Arterial pulsation was palpable beneath the L2 electrode. The electrode impedance and cable connection were checked, and the pulse wave-like artifact diminished after repositioning the L2 electrode. Thereafter, all four traces showed the same EEG pattern as the burst-suppression (Fig. 1b). The PSi, a DOA index calculated by SedLine® Brain Function Monitoring, did not remarkably change. No electrolyte imbalance or hypoglycemia (195 mg dL^−1^) was observed. Arterial blood gas analysis showed an acceptable level of oxygenation. Four days after the second operation, a full recovery, without neurological deficits, was confirmed following extubation.

Numerical values recorded by PRM-7000 and EEG data were exported from SedLine® using the European Data Format and analyzed offline using MATLAB (Mathworks, Natick, MA, USA). Details of the four EEG channels, ECG, and plethysmography before and after the electrode replacement are shown for two 10-s samples (left and right images) in Fig. 1c. To show time locking between the slow-wave EEG and ECG, the EEG signal was first filtered using a linear-phase low-pass filter (cut-off frequency 1.5 Hz), then the Hilbert transform was applied to obtain the analytic signal and the instantaneous frequency was expressed as the digital derivative of the phase of the analytic signal. Finally, the instantaneous frequency curve was smoothed using a moving average over a 4-s window. The instantaneous frequency of the low-frequency component of L2 coincided with the heart rate due to the pulsation artifact and was separated after electrode replacement (Fig. 1d).

## Discussion

This case shows that arterial pulsation can cause an artifact, resulting in anesthesia EEG misinterpretation. The single EEG trace with a regularly occurring slow-wave synchronously with the patient’s pulse was noticeable. Usually, signal contamination in EEG-derived anesthesia monitors is automatically filtered using proprietary software. The current case shows that removing such artifact is impossible due to automatic filtering from the EEG, which is used in the specific processed-EEG monitor, SedLine® Brain Function Monitoring. Moreover, the fact that the PSi value did not change after electrode replacement suggests the PSi calculation algorithm’s robustness. Common artifactual noise, such as frontal muscle contraction or electrocautery, affects several EEG leads because they are not generated within a limited area [5, 6], whereas a pulse wave derivative can be recorded from a single electrode if placed just over the artery. Other possible etiologies of the focal electrical activities that occur in frontal, pathological lesions or motor unit potential have also been suggested. A misinterpretation of EEG-derived index caused by epileptiform activity has been recorded under sevoflurane anesthesia [7]. A cerebrospinal fluid pulsation of frontal pseudomeningocele could also cause an artifact similar to an ictal EEG discharge [8]. If a single motor unit potential is regularly firing under the EEG electrode, spiky electromyograph artifacts are visible overlying EEG [9]. Concurrent contraction of several motor units, including larger motor units, may no longer show a periodic wave [10].

The case-patient showed no seizure-like phenomena and epileptiform activity was not seen in other leads. The pulse wave-like interference in EEG was observed at the heart rate frequency (i.e., 64 cycles min^−1^ or 1.1 Hz, approximately) and occurred only in the L2 electrode. The SedLine® is a distinct DOA monitor with four active EEG leads to collect and process signals from the bilateral frontal lobe. The manufacturer’s manual recommends that the L2 and R2 electrodes be applied to the hairless region just above the left and right temple, respectively. However, STA is one of the two terminal branches of the external carotid artery, and the level and size of these terminal branches differ. Still, the anterior branch, which commonly runs anterosuperiorly, supplying muscles, pericranium, and skin of the lateral frontal area, could be beneath the L2 or R2 electrode. Checking the STA frontal branch pulsation before the electrodes are applied may diminish excessive noise contamination.

The pulsatile artifact was easily detected during visual diagnosis in this case for two reasons: (1) The irregularly contaminated artifacts are hardly distinguishable in patients with arrhythmia. However, the case-patient did not have an arrhythmia. (2) The background EEG waveforms can create prominent artifacts because of burst-suppression or isoelectric EEG pattern. Figure 1d indicates a possible way to detect the pulsatile artifact by comparing the reciprocal of the averaged instantaneous frequency of the low-frequency component of the EEG with the heart rate.

## Conclusions

Numerous clinical conditions, including pulsation artifact, have direct effects on EEG waveform. Sole monitoring of numerical values from DOA monitors may be unreliable; therefore, raw changes in EEG signal should be evaluated to accurately measure the DOA.

## Data Availability

The datasets used and analyzed during the current study are available from the corresponding author on reasonable request.

## Abbreviations

ASA: American Society of Anesthesiologists
DOA: Depth of anesthesia
ECG: Electrocardiogram
EEG: Electroencephalogram
STA: Superficial temporal artery
